# From Reaction Stoichiometry to Life Cycle Assessment: Decision Tree-Based Estimation Tool

**DOI:** 10.1021/acsenvironau.4c00065

**Published:** 2025-07-25

**Authors:** Tim Langhorst, Benedikt Winter, Moritz Tuchschmid, Dennis Roskosch, André Bardow

**Affiliations:** Energy and Process Systems Engineering, Department of Mechanical and Process Engineering, 27219 ETH Zurich, Tannenstr. 3, Zurich 8092, Switzerland

**Keywords:** chemical process design, prospective LCA tool, life cycle assessment (LCA), life cycle inventory (LCI), decision trees

## Abstract

Decision-making during the early stages of research and development (R&D) should be informed by both economic and ecological perspectives. While early stage cost assessments are well established, life cycle assessment (LCA) is still largely descriptive but should expand to a more prospective tool for early assessing the ecological effects of future processes. Chemical processes should be first assessed as early as when only the reaction equation is known. Our previous comparison of estimation methods based on the reaction equation identified three requirements to foster early stage LCA: (1) estimate inventories rather than final impacts to ensure flexibility, (2) distinguish between processes, as single values cannot reflect the variety of chemical processes, (3) provide a measure of uncertainty. In this publication, we propose regression trees to estimate key inputs for industry-scale life-cycle inventories of chemical processes directly from the underlying reaction equation. In detail, the regression trees yield the raw materials’ impact, the direct greenhouse gas (GHG) emissions in CO_2_eq, and the demands for electricity, steam, natural gas, cooling water, and process water. The regression trees outperform the current best available proxy values and provide inventory information that is as accurate as cost estimates. Thus, our work enables decision-makers to consider environmental aspects with the same level of accuracy as costs projections.

## Graphical abstract

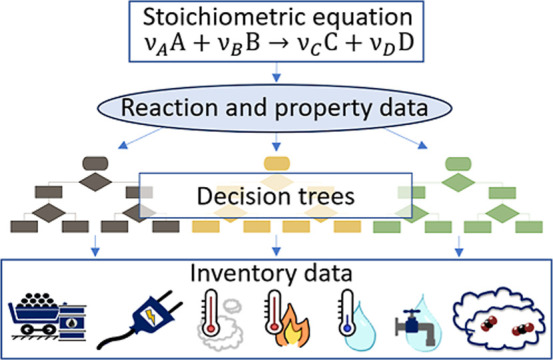

## Introduction

Prospective life cycle assessment (LCA) plays a key role in informing early stage decision-making by evaluating the environmental impacts of future chemical processes. The prospective case of the standardized and holistic LCA approach models and assesses an emerging technology at an early stage of development (e.g., at lab scale) at a later stage of development in the future (e.g., industry scale).^1–4^ Conducting LCAs as early as possible is crucial to incorporate sustainability considerations into the design and development of new chemical processes, because at later stages changes become very costly.^5^ However, at this early stage, the information available about a process is often limited to reaction equations, restricting the quality of the LCA and its usefulness for decision-making.^6,7^ Furthermore, the environmental impacts of background processes connected to the assessed process are often of low quality or unavailable, as only a small fraction of all chemicals produced worldwide is covered by LCA databases.^8^ In both early stage assessments and missing background data situations, LCA primarily relies on stoichiometry-based estimation methods and proxies to estimate potential environmental impacts.^9^ Such proxy values are usually derived from averages for a set of chemical processes and are commonly applied in life cycle inventory databases.^8,10^

In a previous study, we compared existing stoichiometry-based methods to estimate the inventory data of chemical processes.^11^ These methods base estimations of raw material demands on the stoichiometric equation^9,12^ and optionally combine them with default proxies for the yield and energy demands.^13–15^ We highlighted the potential for enhancing those stoichiometry-based estimation methods and suggested an improved method by combining the yield estimation suggested by Geisler et al.^14^ with the average of the process energy demands calculated by Kim and Overcash.^13^ However, we found that single proxy values cannot adequately capture the variety within chemical processes.^11^ A differentiation of the processes is expected to enhance the prediction accuracy of estimation methods. A promising avenue to realize this differentiation are machine learning models.

Available machine learning models for LCA use mixed integer programming,^16,17^ group contribution methods,^18^ or artificial neural networks to directly predict the environmental impacts of target molecules.^19–21^ Since these molecule-based approaches inherently neglect all process and pathway information, process-specific machine-learning models have recently been developed using both molecular and process descriptors to predict environmental impacts.^22–24^

These machine learning models quickly estimate the environmental impacts of new processes and thus are a promising addition for the LCA practitioner’s toolbox. While the current models focus on aggregated cradle-to-gate impacts,^25^ directly estimating the inventory data can increase transparency and interpretability.^9,26,27^ In contrast to estimating environmental impacts, estimating the inventory data, at the level of technical flows, improves the understanding and adaptability of the assessment results:

1 Data is easy to interpret and cross-check with process experts. Consequently, energy scenario analysis and sensitivity checks can be performed based on the estimated technical flows.
2 Technical flow data provides the basis for generating gate-to-gate inventory data which can be used independently from the definition of system boundaries, approaches for solving multifunctionality, the choice of attributional or consequential LCA, Life Cycle Impact Assessment models, and other methodological choices.
3 Technical flow data can be used modularly, e.g., if the raw material demands of a process are known, but heat or electricity demands are unavailable to the LCA practitioner.
4 Input data can easily be refined, e.g., when new information becomes available for single technical flows.

With these advantages, estimated inventories can also enhance the quality of life-cycle inventory (LCI) databases by addressing data gaps resulting from confidentiality concerns or other limitations.^8,11^

Currently, methods are missing to estimate process-specific inventory data from information that is available at early stages of research. The methods should be transparent and easy to apply for practitioners. Decision trees offer this transparency by grouping similar processes by sequential splits of the data.^28^ The sequential splits capture complex interdependencies between input features, while each split is performed based on a simple “yes” or “no” decision.

The concept of decision trees has already been applied in prospective LCA studies. For instance, Karka et al.^29^ employed decision trees to estimate environmental impacts for biobased processes, requiring information from laboratory and early conceptual design stages. However, even the reduced set of input features requires information about the number of solvent types, maximum temperature, and the number of processing steps, which may not be available at an early research stage.

Zhao et al.^30^ applied decision trees to estimate missing elementary flow data for life cycle assessment. Their method identifies and estimates nonzero elementary flows if less than 10% of all elementary flows are missing. However, the authors acknowledge that substantial research is still needed for practical application as the use case of this study was rather artificial, and the exact number of nonzero flows remains unknown in practice.^30^ Pereira et al.^31^ used separate decision trees for each design stage to predict steam demands for batch processes. The classification achieved by both studies (i.e., “low”, “mid”, and “high”) is helpful for initial comparisons but lacks the quantification required for effective early stage decision-making. Decision trees can be used for regression tasks instead of classification to overcome this limitation.

This paper introduces decision trees to estimate important information for chemical processes required to create Life Cycle Inventories: raw materials’ global warming impact, direct GHG emissions in CO_2_eq, and demands for electricity, steam, natural gas, cooling water, and process water. The trees are trained for regression and thus provide specific values and the mean absolute error (MAE) as a measure of uncertainty.

All input data for the trees is derived from the reaction stoichiometry and simple thermophysical data available at a very early research stage, even before first lab experiments are conducted. The trees are trained on inventory data of 409 organic chemical processes based on the Process Economics Programme (PEP) yearbook.^32^ We share the decision trees in a toolbox, including input and output files and a Python code to use the trees. Additionally, we provide short versions of the decision trees to enable manual calculations.

## Methodology

We train decision trees to estimate process-specific energy and utility demands from information available at early research stages. Furthermore, the decision trees provide a coefficient to scale the raw material demands based on the reaction stoichiometry and an estimate for the direct emissions of greenhouse gases. All output data can be estimated based on data available from the stoichiometric equation without knowledge about the process design or reaction conditions. Decision trees are interpretable supervised learning algorithms used for classification and regression tasks.^33^ When predicting LCI data on the level of technical flows, the target values are continuous and thus enable regression. The regression tree algorithm builds “if-then-else” rules to split data into separate nodes, based on the provided input features. Multiple splits in sequence result in a tree-like structure. Features can be categorical, such as whether a side reaction exists, or continuous values, such as boiling points.

In the following, we discuss (1) the training data used for this study, (2) the LCA method and assumptions for this study, (3) the features selected as inputs for the decision trees, and (4) the design and fitting approach of the trees. Subsequently, we discuss (5) the performance indicators and model validation approach.

### Training Data

A large set of detailed chemical gate-to-gate inventory data (e.g., energy, utility, and raw materials) derived from the Process Economics Programme (PEP) yearbook^32^ is available from our previous studies.^11,34^ The PEP yearbook contains validated process reports with information about the required amounts of raw materials and utilities, such as steam, natural gas, electricity, and water. It covers a wide range of chemicals, including platform chemicals, intermediates, and consumer chemicals. The data set is much larger than the data used for calculating the proxy values currently used in Life Cycle Inventory databases. The PEP data is developed for techno-economic assessments and is based on company reports, patents, process analyses, and simulations and is a well-established source for techno-economic process information in industry.^35^ We adjusted the data to reflect the requirements of LCA, e.g., by closing the mass balances, and added the related stoichiometric equations to enable the use as training data.^11,34^

For this work, we focus on processes for organic reactions with chemically well-defined inputs and exclude biochemical and electrochemical reactions. This focus is necessary as the number of processes for inorganic (33), biochemical (15) and electrochemical (9) reactions does not allow accurate training of decision trees for these groups of reactions. The remaining data set contains 409 processes producing 148 unique main products, ranging from platform chemicals such as butadiene, ethylene, or methanol to intermediates such as cyclododecatriene or isopropanol and finally to consumer chemicals such as butylated hydroxytoluene or pyromellitic dianhydride (see Supporting Information Table S1). The data set covers a wide range of global warming impacts (GWIs) (Figure 1) and molar masses, from 26 $\frac{g}{mol}$ for acetylene to 270 $\frac{g}{mol}$ for alachlor, a herbicide banned in the European Union. The GWI reflects the global warming caused by all greenhouse gases emitted during the production of a chemical and is calculated by multiplying the overall emitted amount of each greenhouse gas with its distinct global warming potential over a time horizon of 100 years (GWP100).

**Figure 1:**
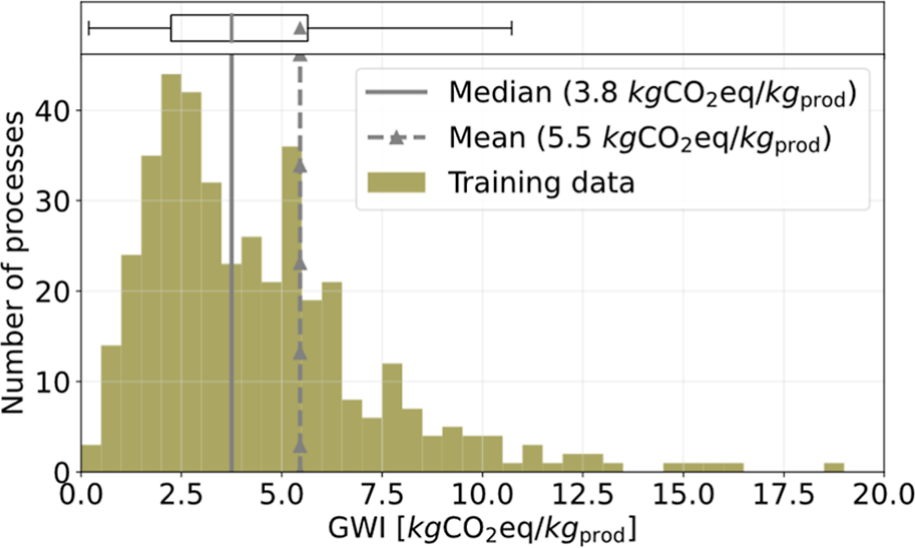
Distribution of the overall GWI (cradle-to-gate, system expansion for valuable byproducts) of the training data as boxplot and histogram (bin width: 0.5), with median (gray line), mean (dashed line and triangle). The GWIs of the processes for the following products are out of the shown range but are included in the training data: hexamethylenediamine, vinylidene fluoride, tetrafluoroethylene, and hexafluoropropylene.

### LCA Methodology

We trained seven separate decision trees for estimating the following gate-to-gate outputs: the demands of (1) electricity, (2) steam, and (3) natural gas in MJ, the demands of (4) cooling water and (5) process water in m^3^, (6) the direct greenhouse gas emissions in CO_2_eq, and (7) the raw materials coefficient (*C*_rm_). The outputs (1) to (5) reflect technical flows and thus can be used to build life cycle inventories applicable to all impact categories. Here, we use the global warming impact (GWI) as an example to enable comparison across all outputs and show how the single outputs can be combined to assess environmental impacts. The GWI was chosen as it is usually a key criterion for early stage decision-making and is categorized in the highest recommendation level for impact factors in the ILCD handbook.^36–38^

The GWI impact category is also the most comprehensive since the direct greenhouse gas (GHG) emissions from the processes are well-represented in the training data, making them suitable for model training (output (6)). In contrast, data on emissions of other chemicals are scarce in the training set. Thus, we decided to only estimate the direct emissions of GHGs. For all other direct chemical emissions, decision trees could theoretically be trained. However, a larger and more detailed data set would be required to achieve predictions of comparable quality to those for GHG emissions. Thus, we recommend using generic emission factors as suggested by Jiménez-González et al., Geisler et al., or Hischier et al. to reflect direct emissions relevant to other environmental impacts.^14,15,39^ Langhorst et al. provide an overview of those emission factors and give recommendations depending on the intended use case.^27^

Furthermore, we estimate the required amount of raw materials (7) to complement the inventories and estimate the overall GWI of the processes. The raw materials coefficient (*C*_rm_) is a dimensionless factor that quantifies how much of the GWI of the raw materials is accounted for through the stoichiometric use of reactants in the process (eq 1). This factor accounts for reaction yield, potential excess of reactants, and the potential use of additional auxiliaries, such as solvents. Thus, this factor streamlines the calculation of the overall raw materials’ GWI from the reaction equation without the need to know the yield for each reactant and the reaction and separation conditions. While this factor is named *C*_rm_ to avoid confusion with the yield, it serves as an alternative to a generic proxy yield for scaling the stoichiometric material demands to generate inventories. However, similar to a generic proxy yield, raw material demands calculated from *C*_rm_ should be used with caution, particularly when calculating other impact categories, because generic proxies and the *C*_rm_ provide only one value for all reactants in a given reaction.

1 $$C_{rm}=\frac{\sum {GWI}_{\;reactants,stoichiometry}}{\sum {GWI}_{raw\;materials,finalprocess}}=\frac{\sum \left({GWI}_{i}\times \frac{m_{i,stoich}}{m_{P,stoich}}\right)}{\sum \left({GWI}_{i}\times \frac{m_{i}}{m_{P}}\right)+\sum \left({GWI}_{j}\times \frac{m_{j}}{m_{P}}\right)}$$

GWI_i/*j*_ is the mass-specific global warming impact of reactant *i* respective auxiliary *j*, *m*_*i*,stoich_ is the mass of reactant *i* as calculated from the stoichiometric coefficient, *m*_*i*_ is the mass of reactant *i* as required in the real process, *m*_*j*_ is the mass of auxiliary *j* required in the real process. All masses are normalized to the mass of the main product (*m*_P_, *m*_P,stoich_).

All estimates reflect the material and energy requirements of the chemical process described by the reaction equation. The results of our comparative study are shown on a cradle-to-gate basis. We apply the functional unit ‘1 kg of main product + side products’ to enable users to use their own allocation approach. We credit produced steam, methane, and electricity via substitution if they are labeled as recovered energy in the PEP yearbook to determine the net energy demands of the process.

The decision trees for the raw materials coefficient and the direct emissions have been trained on the GWI calculated with the life cycle impact assessment method ReCiPe­(H)­V1.13.^40^ We chose ReCiPe­(H)­V1.13 to stay consistent with previous studies using the same data set.^11,41^ The impacts of the raw materials and energy carriers are taken from the ecoinvent database 3.5 ^42,43^ using “cut-off by classification” and global market mixes, if available, to ensure comparability to our previous study on estimation methods.^11^ When calculating the raw materials coefficient, we assume that (i) oxygen, (ii) methane, and (iii) the combination of carbon monoxide and hydrogen will be provided to the final process as (i) air, (ii) natural gas and (iii) synthesis gas. The direct emissions are calculated from the PEP yearbook by closing the mass balances and assuming losses are treated according to Doka and Kätelhön et al.^34,44,45^

To calculate the overall cradle-to-gate environmental impacts, here represented by the GWI, from the decision trees, the stoichiometric impact of the reactants is divided by the raw materials coefficient (*C*_rm_) and the estimated demands *D*_*x*_ are multiplied with their corresponding impacts, here GWI_*x*_ (eq 2). This calculation allows practitioners to choose their own scenarios, e.g., for energy and water.

$${GWI}_{estimated}=\frac{\sum \left({GWI}_{i}\times \frac{m_{i,stoich}}{m_{P,stoich}}\right)}{C_{rm}}+{GWI}_{directemissions}+D_{electr}\times {GWI}_{electr}+D_{heat,{CH}_{4}}\times {GWI}_{heat,{CH}_{4}}+D_{heat,steam}\times {GWI}_{heat,stream}+D_{cooling}\times {GWI}_{cooling}+D_{water}\times {GWI}_{water}$$ 2

The overall quality of the GWI prediction improves with a cleaner energy scenario, as the contribution of the energy demands decreases compared to the raw material demands. The raw material demands can be predicted more accurately based on the reaction equation than the energy demands, which are largely affected by later process design.

Please note that the technical flows estimated by the decision trees are agnostic to the energy scenario. The choice of an energy scenario only becomes relevant when combining and comparing the individual outputs of the decision trees on the level of environmental impacts. In that case, the contribution of the energy demands to the overall GWI depends on the GWI assumed for the energy inputs. For example, a supply of low GHG emission electricity, as expected in the future, might compensate for a poor estimate for electricity.^11^ To prevent any overstatement relating to the accuracy of the decision trees, we use an energy scenario with high GWIs, leading to the least favorable accuracy scenario. For this purpose, electricity is assumed to be provided by combustion of lignite in a power plant.^42,43^

### Feature Selection

Our goal is to ensure the applicability of the decision trees in the early stages of research and development (R&D). Thus, only features about the processes that can be derived directly from the reaction equation or from expert opinions are applicable. However, the features must relate to the property predicted by the trees. We identified the most useful features in a prestudy by calculating the feature importance and systematically deleting features that do not improve the overall model performance based on a leave-one-out cross-validation (see Supporting Information Figure S1). The final decision trees are trained using ten molecular features and six reaction-based features (Table 1). The boiling points have been calculated using statistical thermodynamics from COSMO-RS.^46^

**Table 1:** List of Features Used for the Final Decision Trees^a^

feature (group)	feature name	description	minimum value in training set	maximum value in training set	median of training set
molecular features					
boiling points	BP_maxE_	maximum boiling point of all involved reactants	20 K	844 K	351 K
	BP_minE_	minimum boiling point of all involved reactants	20 K	605 K	90 K
	BP_maxP_	maximum boiling point of all involved products	20 K	762 K	402 K
	BP_minP_	minimum boiling point of all involved products	20 K	538 K	370 K
molecular weight	*M*_W_mainP__	molecular weight of main product	26 g/mol	270 g/mol	89 g/mol
key atoms in the reactants	Cl	mol of chlorine atoms in the reactants per mol of product	0 (361)	8	0
	F	mol of fluorine atoms in the reactants per mol of product	0 (403)	6	0
	N	mol of nitrogen atoms in the reactants per mol of product	0 (341)	12	0
	C	mol of nonpi/noncyclic carbon atoms in the reactants per mol of product	0 (22)	16	3
	c	mol of cyclic carbon atoms in the reactants per mol of product	0 (297)	18	0
reaction-based features					
reactants	countReac	number of reactants in the reaction stoichiometry	1	5	2
	stoichioH_2_	mol of H_2_ required as reactants	0 (334)	10	0
products	countPro	number of products in the reaction stoichiometry	1	5	2
	AddSidePro	expected occurrence and separation of additional side products from possible side reactions	0 (=no) (303)	1 (=yes)	0
	water	mass of water [ $\frac{kg}{{kg}_{main\;product}}$ ] formed stoichiometrically	0 (217)	2.1	0
	*X*_mainP_	molar fraction of the main product assuming 100% yield	0.1	1	0.5

a The minimum and maximum values for each feature as used in the final training set indicate the applicability range of this model. In case the minimum value is “0”, the number of “0” in this data set is provided in brackets. Table S1b provides additional information on the 1st and 3rd quartile.

Machine learning models generally have a limited capability to extrapolate, particularly in case the unseen data strongly differs from the training data. Thus, we recommend using the decision trees only for organic reactions within the ranges of the training data provided in Table 1. The trees are not trained for electrochemical or biochemical routes.

### Tree Design and Fitting Approach

We used the decision tree regression model from the scikit-learn module (version 0.23.2) in Python 3.8 to train the decision trees. This model uses a modified version of the CART algorithm to find the best split.^47,48^ Each split partitions the data into two subsets, called nodes, with similar target values by minimizing the variability within both resulting nodes. To determine the best feature and location for the next splits, we let the model minimize the mean absolute error (MAE). Based on this criterion, the predicted values are less prone to outliers because they are based on the median of their node, while the algorithms for all other minimization criteria use the mean.^48^ For each possible node, the MAE_node_ is calculated (eq 3). The splitting algorithm searches for the split minimizing the weighted sum of both nodes resulting from a split.

3 $${MAE}_{node}=\frac{1}{n_{node}}\sum_{y\in node}\left|y-median\left(y_{node}\right)\right|$$

To avoid overfitting, the two most efficient and common approaches for decision trees are (1) to impose a minimum number of samples in a single node and (2) to limit the maximum depth of the decision trees.^47,49^ The depth describes the maximum number of splits in sequence within the decision tree. Based on a prestudy, we limited the minimum number of samples in each node to 4, which is approximately 1% of all samples. The decision tree should be as deep as necessary to distinguish between data points and as short as possible to avoid overfitting. The depth was, therefore, set to 7, which allows the tree to grow to a maximum of 128 final nodes. With a fixed minimum of 4 samples per node, the possible nodes could cover all processes within the training data set. The tree algorithm does not necessarily result in equal sample sizes in each node. Thus, the actual number of final nodes is expected to be lower. We checked the performance in a prestudy with a reduced training set and found the depth to be appropriate (see Supporting Information “Prestudy”).

### Performance Indicators

Our goal is to enable early stage LCA with a quality comparable to cost estimates, as both costs and environmental impacts should be used in decision-making during R&D. LCA can benefit from decades of experience in quantifying and communicating uncertainties of cost estimates as collected and published by the Association for the Advancement of Cost Engineering (AACE) and used for techno-economic assessments. Thus, we use established performance indicators from cost engineering for our study: the mean absolute error (MAE) and the percentage of processes outside the target range published by the AACE.^50,51^ This comparison basically answers the question: “Can we predict LCI data within the same accuracy ranges as cost data?” The mean absolute error is also used to train the decision tree algorithm and is described in eq 3. The AACE proposes an accuracy range of −50% to +100% for cost estimates at this early research stage.^50,51^ For costing, this accuracy range covers approximately 90% of all processes.^50,51^ We aim to achieve comparable accuracy for LCA to enable consistent decision-making in cost estimates and LCA.

The results shown in the following are based on unseen data from a leave-one-out cross-validation. A prestudy showed that this approach reflects the performance of the final decision trees on an independent test set (see Supporting Information Table S2). This prestudy showed that 369 processes were sufficient for training decision trees that outperformed generic proxy values on an independent test set of 40 processes.

## Results

Performance of the decision tree regression model.

Our goal is to predict the most common technical flows for life cycle inventories from a reaction equation and improve the accuracy compared to existing proxy-based approaches. To test the overall performance of our prediction, we combine the results of the individual decision trees for the raw materials’ impact, the direct emissions, and the utilities (heat from steam and natural gas, electricity, cooling water, and process water) according to eq 2. We use the data from the PEP yearbook to benchmark the estimated results, as the best data available to us. However, we want to highlight that all LCA data can have high degrees of uncertainty and variability and that our results can only be as accurate as the process data provided in the PEP yearbook. Figure 2 compares the accuracy of the decision trees in predicting the GWI to the best performance achieved by default proxy values.^11^ The proxy approach applies a generic demand for heat and electricity to all processes.^13^ Furthermore, we assume one of two generic yields, depending on whether side products occur or not.^14^ The proxy values used for this comparison have been identified as the most suitable in our previous study.^11^ Thus, these values reflect the highest accuracy that can be reached using established default values. For a fair comparison, we used the same LCA method and assumptions for both estimation methods.

**Figure 2:**
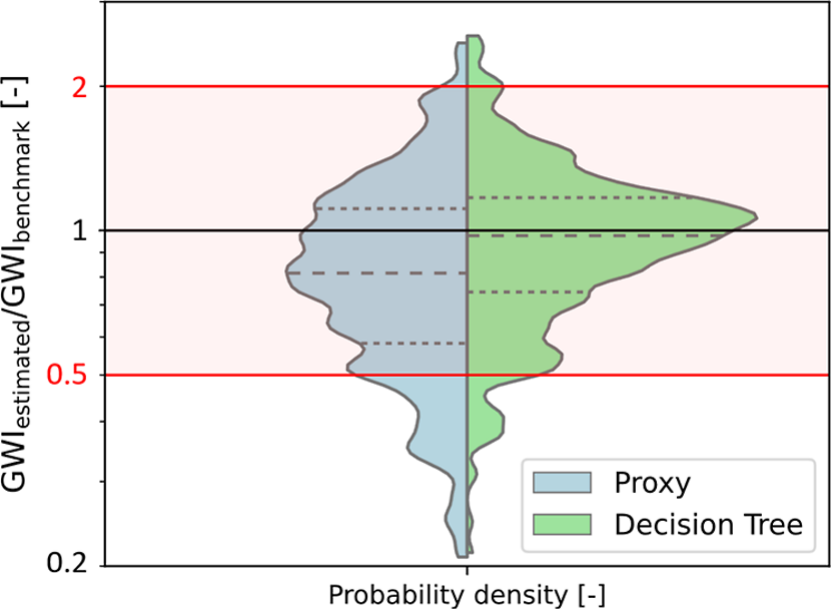
Violin plot comparing the overall GWI estimation performance when using the best available proxy values^11^ for each technical flow (left) with the new decision trees (right). Results reflect cradle-to-gate system boundaries in both cases. The performance is measured using a leave-one-out cross-validation. The sample lies on the black horizontal line if the estimated GWI equals the benchmark GWI (perfect prediction). Below this line, the GWI is underestimated; above, it is overestimated. The logarithmic scale shows the equal relevance of under and overestimating by a factor of 2 (red lines). The red area between those red lines (0.5 to 2) provides orientation between Figures 2 and 3. The dashed lines in the violin plots mark the quartile ranges and the median of the data sets.

Default values cannot reflect the full range of chemical processes due to the high variability of chemical reactions and process setups. Such variability can lead to substantially different demands for utilities like heat and electricity.^11^ Decision trees can reflect such variability by offering distinct values instead of a single proxy. Combining the results of the decision trees increases the accuracy of prediction compared to combining default proxies. The increase in accuracy is visible in two effects: First, we see a shift of the mode and median toward 1, reflecting a perfect prediction (from 0.82 to 1.06 and from 0.81 to 0.98). Second, the more narrow distribution indicates an improved precision. Using the decision trees, the second and third quartile of all processes are estimated within −26% to +18% to a perfect prediction, compared to the method using default proxies where the second and third quartile are predicted in a range of −43% to +12% to a perfect prediction. Furthermore, only 11% of all processes are estimated outside the target range from −50% to +100% (see Supporting Information Table S3).

The mean absolute error of the separate utility estimates reduced by 8–20%, resulting in an improvement of 15% for the sum of all utilities (see Supporting Information Table S4). By reducing the mean absolute error, the decision trees also reduce the number of processes estimated outside the AACE target range by 17–31% resulting in an improvement of 19% for the sum of all utilities (see Supporting Information Table S4).

The overall GWI of the raw materials can be predicted with a high level of accuracy, reflecting the strong correlation of the reaction equation and the final raw material demands. The best available proxy-based method distinguishes between processes where side reactions occur and those without side reactions. This distinction already leads to a high level of accuracy with the median at 1.022 [ $\frac{{GWI}_{estimated}}{{GWI}_{benchmark}}$ ] and the second and third quartile of the processes estimated between −15% and +11% above or below 1 (perfect prediction). The decision trees further increase the accuracy of the prediction with the median at 1.003 [ $\frac{{GWI}_{estimated}}{{GWI}_{benchmark}}$ ] and the second and third quartile estimated between −7% and +6% to a perfect prediction. This increase in accuracy becomes particularly relevant as the GWI of raw materials is often the major contributor to the overall GWI.^11^ The required raw materials were calculated using the raw materials coefficient *C*_rm_ and thus may not present other impact categories as good as the GWI. Nevertheless, the *C*_rm_ may still better represent the required amount of raw materials than a generic yield.

The utility demands are affected by the whole process design and become known only at a late stage of process development.^52^ Therefore, the information derived solely from the reaction equation cannot fully reflect the utility demands. Consequently, the distribution remains relatively wide compared to the raw materials’ GWI (Figure 3). Thus, the decision trees improve the predictions even more. By providing estimates specific to reactions, the decision trees shift the mode and median toward 1 and reduce the variance, indicating they effectively capture the diversity of processes.

**Figure 3:**
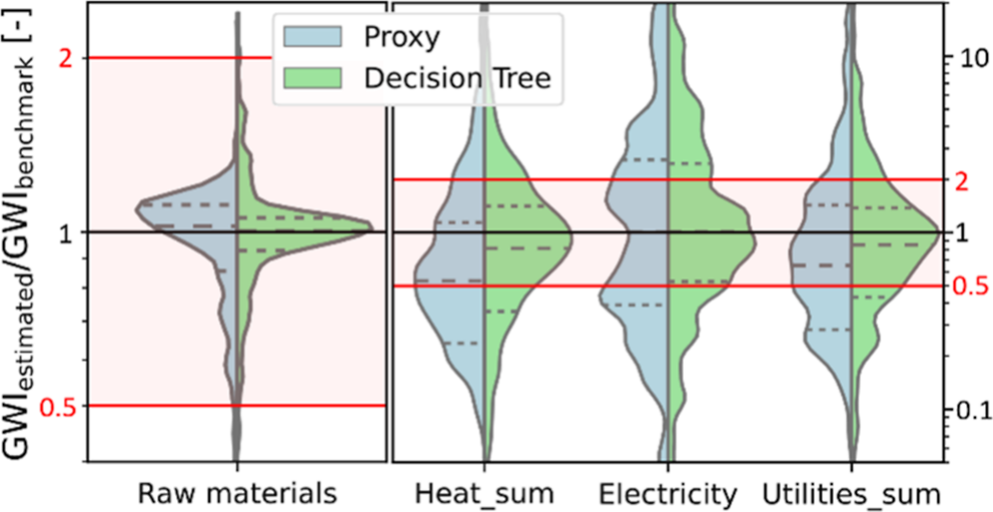
Violin plots comparing the estimation performance of the best available proxies (left) with the new decision trees (right) for estimating the aggregate GWI of the raw materials, the total heating demands in MJ, electricity demand in MJ and the aggregate GWI of all utilities. Please note the different ranges on the *y*-axis. For each utility, the results are equal on the inventory flow level and on the impact level, as both the estimated value and the benchmark value are multiplied with the same characterization factor. The performance is measured using a leave-one-out cross-validation. The sample lies on the black horizontal line if the estimated value equals the benchmark process data (perfect prediction). Below this line, the technical flow is underestimated; above, it is overestimated. The logarithmic scale shows the equal relevance of under and overestimating by a factor of 2 (red lines). The red area between those red lines (0.5 to 2) provides orientation between the two *y*-axis scales and simplifies the comparison to Figure 2. The dashed lines in the violin plots mark the quartile ranges and the median of the data sets.

In particular, we observe a strong shift of mean (0.66 to 0.88) and mode (0.59 to 0.89) from the lower end of the target range to a perfect prediction for the heating demands (steam and natural gas combined, see Figure 3 “Heat_sum”). In the case of electricity demand, the median resulting from the proxy use was already close to a perfect prediction (1.00). However, we observe a bimodal distribution characterized by peaks at both ends of the target range (0.44—1.5). This bimodality is due to two clusters of processes that a single proxy cannot reflect. In contrast, the decision trees result in a single mode (0.86), indicating they can distinguish those clusters. For all technical flows, the trees reduce the interquartile ranges, meaning the prediction accuracies of the middle 50% of all processes are less dispersed compared to the estimates by single proxies. This reduced dispersion is also reflected in the last violin plot, which shows the overall accuracy of all utilities combined with regard to their GWI (see Figure 3 “Utilities_sum”, mode shifted from 0.78 to 0.98). Table S4 lists the mean absolute errors (MEA) and the percentage of processes outside the target range for all estimated technical flows and the direct emissions of GHGs based on the units estimated by the decision trees, e.g., energy in MJ and water in m^3^.

In summary, the decision trees can reflect differences between chemical reactions and thus better reflect the variability in chemical processes. For early stage assessments, the estimated yield (via the raw materials coefficient *C*_rm_), direct GHG emissions, and utility demands enable a distinction between chemical reactions during research and development. Hotspots like a high estimated demand for heat can be discussed with process engineers to raise concerns early on and check potential issues.

However, the uncertainty of the estimated results remains high due to the limited information required for their use and the variability in final process designs, even for the same chemical reactions. Process setups can vary significantly even for existing production routes, including our training data. The mean absolute error provided by the decision trees helps assess the uncertainty of the decision trees but misses further uncertainty arising from the limitations of the data set. Thus, estimates should be regarded critically and improved once better data becomes available during process design. If such more detailed data becomes available, the analysis can and should be refined, e.g., using methods from Parvatker and Eckelman or El-Halwagi.^9,53^ The Low-TRL Guidelines and the ESTIMATE tool summarize and recommend specific methods for each research and development stage.^27,54^ The guidelines are supplemented by multiple worked examples, YouTube videos, and a Web site offering further tools.^55,56^

### Interpretation of the Decision Trees

Decision trees can visualize the decision path and thus enable an understanding of the learned decisions. The trees were trained using a decision tree regression model without prior chemistry or process engineering knowledge. Thus, we want to understand whether the decisions align with such knowledge. In the following, we exemplarily discuss the first layers of the decision tree estimating the raw materials coefficient *C*_rm_ (Figure 4). *C*_rm_ reflects the average yield for all reactants and further accounts for auxiliaries (eq 1). The yield for single reactants may vary from this average depending on which reactants may be provided in excess (see Methodology).

**Figure 4:**
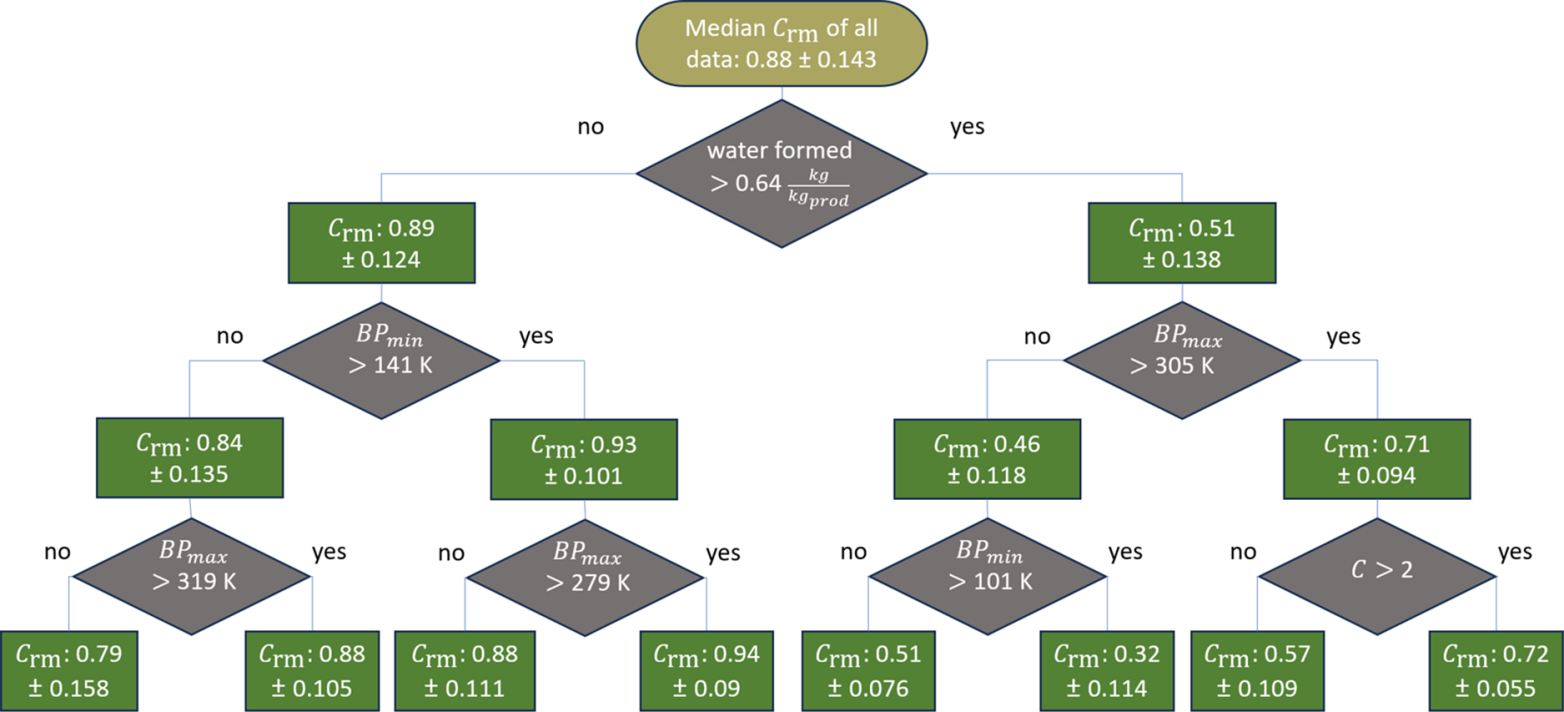
First three layers of the decision tree for estimating the raw materials coefficient *C*_rm_. Without any decision taken (pill shaped box), the median of all data points is at 0.88 [ $\frac{{GWI}_{stoichiometric}}{{GWI}_{benchmark}}$ ] with a mean absolute error of 0.143. The data is split with each decision (diamonds) to minimize the overall mean absolute error. The rectangles represent the median and the mean absolute error of the resulting split groups (nodes). BP represents the normal boiling point of the reactants. Here, the minimum or the maximum of all reactants are used as splitting criteria. *C* represents moles of (aliphatic) carbon atoms involved in the reaction per mole of the main product.

The first decision divides the data set into those processes with a maximum of $0.64\frac{kg\;water}{kg\;product}$ formed, and those processes with higher amounts of water formed. If a high amount of water is formed during the reaction, *C*_rm_ is expected to be only 0.51. This first decision is reasonable, as there is generally a negative correlation in the training data between the amount of water formed and the required material inputs. Water is an additional byproduct increasing the purification efforts and decreasing the overall yield of the final purified product.

If more than $0.64\frac{kgwater}{kg\;product}$ is formed, the next decision is whether the maximum normal boiling point of the reactants is above 305 K or not. This split roughly divides the reactions into those with oxygen included in the organic reactant (here, 10 out of 11 reactions contain alcohols or carboxylic acids) and those in which pure oxygen is provided (true for all 21 processes). Thus, the decision tree distinguishes between oxidative dehydrogenation and dehydration reactions as the reason for water formation. Oxidative dehydrogenation processes are often unspecific, with unwanted side reactions likely to reduce the yield, particularly for alkanes.^57,58^ Furthermore, often an oxidative dehydrogenation, as indicated in the reaction equation, cannot be realized at a large scale, thereby requiring the use of even more unspecific dehydrogenation routes with low equilibrium concentrations and auxiliaries required for catalyst regeneration.^57,58^ Thus, a higher boiling point indicating dehydration processes being used, instead of dehydrogenation, correlates with a lower raw material demand (higher *C*_rm_). This correlation is reasonable, as a high mass ratio of water indicates that the product molecules are relatively small, compared to the number of functional groups. In our case, the average molecular weight of the main product decreases from $98\frac{g}{mol}$ to $61\frac{g}{mol}$ if more than $0.64\frac{kg\;water}{kg\;product}$ is formed. In this case, a high boiling point usually results from functional groups increasing the molecule’s polarity. A possible exception to this rule are ammoxidation processes, as those also result in a high amount of water as a byproduct and can be used for small and large molecules to achive nitriles with a medium *C*_rm_ (44–72% in our training data).^59^

If less than $0.64\frac{kg\;water}{kg\;product}$ is formed during the reaction, the raw materials coefficient *C*_rm_ remains at 0.89. In this case, the next decision is whether the minimum normal boiling point of the reactants is above 141 K or not. By choosing this temperature to split the data, the model incorporates important information: For our data set, the reactants below this boiling point are hydrogen, carbon monoxide, or methane, which are gases used for C1 chemistry. Thus, if the question is answered with “no”, the routes involve C1 chemistry or the reaction with hydrogen. Those reactions often use the gases in excess and are more likely to require auxiliaries (1.16 auxiliaries in average, compared to 0.75 auxiliaries for the other reactions). Furthermore, the reactants for C1 chemistry have a rather low GWI (average: 2.1) compared to the reactants for other reactions (average: 5.1) increasing the effect of additional auxiliaries on the overall GWI. Thus, a decrease of *C*_rm_, as suggested by the decision tree, seems well aligned with industrial practice.

The third decision layer can be summarized by one general rule: A decrease in the raw materials coefficient *C*_rm_ is expected when the boiling points of the reactants are closely aligned. This comparison between highest and lowest boiling point is possible, as the previous decision was based either on the lowest or highest boiling point of the reactants and the current decision layer is based on the remaining. If the boiling points are closely aligned, *C*_rm_ is expected to be lower. This expectation is reasonable, as we can expect the separation to be more challenging. Thus, the separation may require additional auxiliaries and result in product losses if their recovery is not feasible.

One decision does not focus on the boiling points but rather checks whether the reactants have more than two carbon atoms in total, which usually means that the final product consists of more than two carbon atoms. If this is not the case, a lower *C*_rm_ is estimated. Our data shows that the related processes share a low selectivity of the catalysts, resulting in a smaller *C*_rm_ for the process. The side products are recovered and sold for those processes. The small *C*_rm_ reflects a high raw materials demand of the overall process, including all side products within the system boundaries. This example shows the general importance of considering the system expansion approach used with the presented decision trees to estimate the utility and raw materials demands of the overall processes. Product-specific assessments could be achieved by crediting the side products in a subsequent step.

The trees created by the decision tree regression model make decisions that can be explained from a chemical and process engineering perspective even though the required knowledge was not explicitly provided to the model, e.g., the reaction mechanism. This knowledge was derived from other related features combining sequential decisions. Furthermore, the decision trees provide the mean absolute error, reflecting the uncertainty of each node and thus allowing for individual uncertainty assessments.

### Accessibility of Decision Trees as a Toolbox

With this study, we aim to provide a user-friendly approach to estimating missing inventory data in the form of important technical flows. Therefore, the final decision trees are made available in a toolbox, which includes a Python script to use the decision trees and a file for manual user inputs for all feature data (further details in readme file). The required reaction data can be derived directly from the stoichiometric coefficients:

- Amount of water formed during the reaction,
- Amount of hydrogen required for the reaction,
- Total number of products and reactants
- Molar ratio of the main product compared to all products.

The required property data are:

- The boiling points of reactants and products,
- The amount of Cl, F, N, and C atoms in the reactants
- The molecular weight of the main product.

Furthermore, users may indicate whether side-products from side reactions are expected and should be separated during the downstream process. Often, this information is not available at the early stages of research. Thus, we recommend setting this value to 0 (no side-product) per default (see Supporting Information “Additional side products”). In addition to the manual entry of all feature data, the toolbox allows the user to alternatively derive all required feature data directly from the stoichiometric coefficients and the CAS Registry Numbers. To offer this alternative, the script contains a workflow to calculate the reaction-specific features from the stoichiometric coefficients and search property data in the DIPPR 801 database.^60^ This automated search requires access to the DIPPR 801 database.^60^ However, the open-source script in the modular toolbox allows practitioners to add further databases, if required.

In addition to the full decision trees in the toolbox, we offer short visual versions of the trees that can be used manually. The short trees represent the first four decisions of the full trees (see Supporting Information Figures S5–S11). This depth is sufficient to outperform default proxies (see Supporting Information “Additional side products”, and Figures S3 and S4).

The decision trees can be used modularly, e.g., only using those outputs that are not available for the studied process. This modularity allows training additional trees for further outputs or improving specific trees when additional training data becomes available. The toolbox, therefore, allows adding further decision trees in the future. Thus, we want to highlight the limitations of the decision trees that users should consider, and that may be closed in the future:

First, the decision tree for direct emission accounts only for greenhouse gases due to a lack of other information on direct emissions (see LCA methodology). In principle, decision trees can and should be trained for all types of direct emissions once sufficient training data is available.

Second, decision trees may be trained to separately predict the yield and demands for solvents and other auxiliary materials. However, predicting solvents and auxiliary materials in detail is challenging, particularly when only the reaction equation is available. Thus, the raw material coefficient introduced here seems to provide an easy-to-use alternative at early stages of research and development.

Third, the decision trees currently do not capture catalysts due to the lack of training data and confidentiality issues. While involved researchers may provide information on catalysts, the challenge of missing background data on catalysts in databases remains.

## Conclusions and Outlook

Our study introduces accessible and transparent decision trees that enable the estimation of inventories for organic chemical processes at a very early stage of process development by relying solely on information derived from the reaction equation and thermo-physical properties of the reactants and products. The decision trees can be used in screening studies without detailed process design or operating conditions. Seven individual trees were trained to estimate the raw materials coefficient, the direct emissions in CO_2_eq, and the demands for electricity, steam, natural gas, cooling water, and process water.

Compared to the best available proxy values, the decision trees reduce the mean absolute error of the estimates for the overall GWI by more than 20% and the number of processes estimated worse than the target accuracy by more than 40%. The decision trees outperformed each of the default proxies for the technical flows, with the mean absolute error reduced by 8–24% and the number of processes out of the target range reduced by 17–31%. With the trees, LCA can reach similar confidence levels as expected for cost estimates at the same early R&D stage.^51^

The decisions of the trees align with basic chemistry intuition and process design knowledge. Thus, on the one hand, the decisions made can be discussed with process designers. On the other hand, the decision criteria can be useful for chemists when searching for promising reaction equations. In addition to the estimated value, the trees provide the mean absolute error and thus reflect the uncertainty of the estimate. Both, the provided uncertainty measures and the transparency of the decision paths, enable more comprehensible assessments and more thorough discussion of the limitations than default proxies. Still, the decision trees are also limited by the accuracy of their training data, such that stoichiometry-based estimation methods can only provide initial LCA results. Thus, we recommend discussing the uncertainties and limitations with stakeholders early on and checking the decision path provided by the trees. The decision trees are available in a ready-to-use toolbox together with input and output Excel files, and a Python code to eventually fill in missing inputs and run the decision trees. As an alternative to the manual input of the reaction-based and property data, we offer an autofill option requiring access to the DIPPR database.^60^ We thereby reduced the required user input to the CAS Registry Numbers and stoichiometric coefficients. Thus, the toolbox enables the broad use of our decision trees.

Our toolbox allows for the rapid construction of preliminary inventories for industry-scale processes solely from the stoichiometric equation and can be used for processes at the earliest R&D stage as well as for gaps in inventory databases. As the tool calculates each technical flow separately, the flows can and should be adjusted as soon as more detailed information becomes available during the R&D process.

## Supplementary Material



## Data Availability

The trained decision trees and the supporting files are openly available on GitHub at: https://tlanghorst.github.io.

## Notes

Including a list of included chemicals, information about the Prestudy, and short decision trees (PDF)

## Abbreviations

AACE: association for the advancement of cost engineering
CART: classification and regression trees
CAS: chemical abstracts service
DIPPR: design institute for physical properties
GWI: global warming impact
LCA: life cycle assessment
LCI: life cycle inventory
MAE: mean absolute error
PEP: process economics program
R&D: research and development
