# Supplementary material for: From Reaction Stoichiometry to Life Cycle Assessment: Decision Tree-Based Estimation Tool
Source: ACS Environ Au. 2025 Jul 25;5(6):550–60. doi: 10.1021/acsenvironau.4c00065 (PMC12635940; doi:10.1021/acsenvironau.4c00065)
Supplement: Supplementary file 1 [file vg4c00065_si_001.pdf]

Supplementary information:
From reaction stoichiometry to life cycle assessment: Decision tree-based estimation tool

Journal: ACS Environmental Au

Authors

Tim Langhorst, Benedikt Winter, Moritz Tuchschmid, Dennis Roskosch, André Bardow*

Energy and Process Systems Engineering, Department of Mechanical and Process Engineering, ETH Zurich, Tannenstr. 3, Zurich, 8092, Switzerland

*Corresponding author: André Bardow, abardow@ethz.ch

Abbreviations

AACE	association for the advancement of cost engineering (here used for the suggested range in their Cost estimate Guide)[1, 2]
GWI	global warming impact
LOO-CV	leave-one-out cross validation
MAE/mae	mean absolute error
MW	molecular weight

Supplementary information:

Included Chemicals

Table S1: List of all chemicals included in the final training data set. Chemicals used as test data for testing the performance on the reduced training data set are marked (*).

main product	MW $[\frac{g}{mol}]$
1,1-Dichloro-1-fluoroethane*	117
Dodecanedioicacid	230
Butadiene*	54
1,3-Propanediol	76
1,4-Butadiol*	90
1,4-Cyclohexanedimethanol	144
1-Butene	56
1-Hexene	84
Octene	112
2,4-Dimethylbenzaldehyde	134
2,6-Diethylbenzeneamine	149
2,6-Dimethylphenol*	122
2,6-Bis(1,1-dimethylethyl)-phenol	206
2-Ethyl-1-hexanol	130
2-Methylpyridine	93
4-Methyl-1-pentene*	84
Acetaldehyde*	44
4-Hydroxyacetanilide	151
Acetic acid*	60
Aceticanhydride	102
Propanone	58
2-Propanol*	60
Ethine	26
Acrolein	56
2-Propenamide	71
Acrylic acid*	72
2-Propenenitrile	53
Hexanedioic acid*	146
Alachlor	270
2-Propen-1-ol*	58
3-Chloropropene	77
Aniline	93
Anthraquinone	208
Benzene	78
Benzoicacid	122
4,4'-(1-Methylethylidene) bisphenol	228
2,6-Bis(1,1-dimethylethyl)-4-methylphenol	220

main product	MW $[\frac{g}{mol}]$
2-Pyrrolidon	85
gamma-Butyrolactone	86
Carbofuran	221
Chloroaceticacid	95
Chloroacetylchloride	113
Chlorobenzene	113
1-Chloro-1,1-difluoroethane*	101
CH3Cl	50
2-Chloro-1,3-butadiene*	89
Chlorothalonil	266
Isopropylbenzene	120
1,3,5-Triazine-2,4,6(1h,3h,5h)-trione	129
1,5,9-Cyclododecatriene	162
Cyclohexane	84
Cyclohexanol	100
Cyclohexanone	98
Cyclohexanoneoxime	113
Diisopropylether	102
Dimethylcarbonate*	90
Dimethylether	46
Dimethylsulfoxide	78
1,4-Benzenedicarboxylicaciddimethylester	194
1-Methyl-2,4-dinitrobenzene	182
Dipropylamine*	101
Diphenylcarbonate	214
n-Phenyl-benzeneamine	169
Glycol	62
(Chloromethyl)-oxirane	93
Ethanol	46
Ethanolamine*	61
Ethylacetate	88
2-Propenoicacidethylester	100
2-Hydroxypropanoic acid ethylester	118
2-Ethoxy-2-methyl-propane	102
Ethylbenzene*	106
Ethene*	28

**main product**	**MW** [$\frac{g}{mol}$]
1,3-Dioxolan-2-one*	88
1,2-Dichloroethane*	99
2-Butoxyethanol	118
Ethoxyethanol	90
Ethyleneglycoltert-butylether	118
Ethyleneoxide	44
1,2-Diaminoethane	60
Formaldehyde	30
1,1,2,3,3,3-Hexafluoro-1-propene	150
1,6-Hexanediamine*	116
Isobutene*	56
Nonanal	142
Hydroquinone	110
Isobutylbenzene	134
Isononylalcohol	144
Isophoronediisocyanate	222
1,3-Benzenedicarboxylicacid	166
1,3-Benzenedicarbonitrile	128
1,3-Benzenedicarbonyldichloride	203
Isoprene*	68
1-Amino-2-propanol	75
2-Chloropropane	79
1,2-Epoxypropane*	58
Maleicanhydride	98
3-Aminophenol	109
1,3,5-Triazine-2,4,6-triamine*	126
Methanol	32
Propene*	42
Methylacrylate	86
Carbonochloridic acid methylester	95
Methylformate	60
4-Methyl-2-pentanol	102
4-Methyl-2-pentanone	100
Isocyanatomethane	57
Methanethiol	48
Methylmethacrylate	100
Dimethylamine	45
Methyl-t-butylether	88
3-Methylaniline	107

**main product**	**MW** [$\frac{g}{mol}$]
n,n-Diethyl-benzeneamine	149
n,n-Dimethylacetamide	87
n,n-Dimethylaniline	121
Dimethylformamide*	73
1-Butanol*	74
2-Propenoicacidbutylester*	128
1-Isocyanatobutane	99
1-Butylamine	73
Butanal*	72
Nitrobenzene	123
n-Methyl-2-pyrrolidinone*	99
1-Chloro-4-nitrobenzene	158
1-Chloro-4-methylbenzene	127
Peroxyaceticacid	76
Phenol	94
4-Methylstyrene	118
1-Pentanol	88
Propylenecarbonate	102
Propyleneglycol	76
4-(1,1-Dimethylethyl)-phenol	150
4-(1,1,3,3-Tetramethylbutyl)-phenol	206
1,4-Benzenedicarboxylicacid*	166
1,4-Dimethylbenzene	106
1,2,4,5-Benzenetetracarboxylicanhydride	218
Styrene*	104
Terephthaloylchloride	203
Tetrafluoroethene	100
THF	72
1,4-Butanediamine	88
1,3-Diisocyanato-2-methylbenzene	174
Trichloroethene	131
Trimellitic-anhydride	192
Vinylacetate*	86
Chloroethene	63
1,1-Dichloroethylene	97
1,1-Difluoroethene	64

Table S1b: List of features used for the final decision trees. The minimum and maximum values for each feature as used in the final training set indicate the applicability range of this model. In case the minimum value is ‘0’, the number of ‘0’ in this data set is provided in brackets.

Feature (group)	Feature name	Description	Minimum	Maximum	1st Quartile	Median	3rd Quartile
**Molecular features**							
Boiling points	BP_{maxE}	Maximum boiling point of all involved reactants [K]	20	844	254	351	412
	BP_{minE}	Minimum boiling point of all involved reactants [K]	20	605	90	90	240
	BP_{maxP}	Maximum boiling point of all involved products [K]	20	762	373	402	473
	BP_{minP}	Minimum boiling point of all involved products [K]	20	538	188	370	373
Molecular weight	MW_{mainP}	Molecular weight of main product [g/mol]	26	270	60	89	118
Key atoms in the reactants	Cl	Mol of chlorine atoms in the reactants per mol of product	0 (361)	8	0	0	0
	F	Mol of fluorine atoms in the reactants per mol of product	0 (403)	6	0	0	0
	N	Mol of nitrogen atoms in the reactants per mol of product	0 (341)	12	0	0	0
	C	Mol of non-pi/noncyclic carbon atoms in the reactants per mol of product	0 (22)	16	2	3	4
	c	Mol of cyclic carbon atoms in the reactants per mol of product	0 (297)	18	0	0	6
Reaction-based features							
Reactants	countReac	Number of reactants in the reaction stoichiometry	1	5	2	2	3
	$stoichioH_2$	Mol of H_2 required as reactants	0 (334)	10	0	0	0
Products	countPro	Number of products in the reaction stoichiometry	1	5	1	2	2
	AddSidePro	Expected occurrence and separation of additional side products from possible side reactions	0 (=no) (303)	1 (=yes)	0	0	1
	water	Mass of water $[\frac{kg}{kg_{main\ product}}]$ formed stoichiometrically	0 (217)	2.1	0	0	0.24
	X_{mainP}	Molar fraction of the main product assuming 100% yield	0.1	1	0.33	0.5	1

Pre-study

We compared the performance of the decision trees based on a leave-one-out cross validation (LOO-CV) and a test set to ensure that the LOO-CV reflects the performance of the decision trees with all data used for training on external data. The inventory data of 474 processes from a previous publication was filtered to exclude inorganic reactions, bioprocesses and electrochemical processes.[3,4] For those processes there was not enough data for training separate decision trees. Thus, this study focuses on organic reactions, for which 409 processes were identified.

First, we excluded a random set of 40 processes (from 409, equals 10%) for testing. Second, we used the remaining 90% of the data for training the model and performed a LOO-CV. Each sample of the training data is left out once when training the decision trees and used to validate the performance of the resulting trees. The LOO-CV was used to check the decision tree depth and to reduce the features. Afterwards, we trained preliminary decision trees on the reduced training data (369 processes) to use for testing.

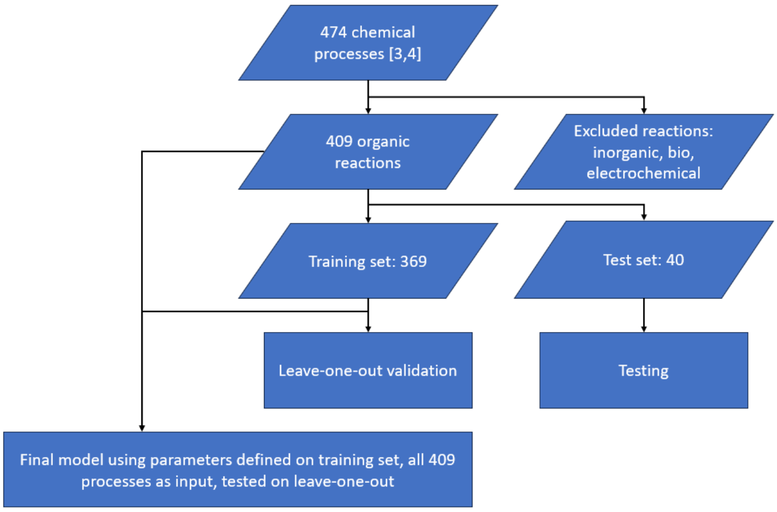

Figure S1 Workflow for managing the data sets. 10 % of the available data was used as a separate test set to check whether the results from a LOO-CV were comparable to those obtained from a test set. As both results are comparable, the final model was tested with the leave-one-out approach, allowing the use of all 409 processes as input.

Table S2: Comparison of the validation trees trained on 369 data points. The performance is calculated based on the test set (remaining 40 processes) and on LOO-CV.

Measure	Test set (40)		LOO-CV (369)	
	MAE	Out of AACE	MAE	Out of AACE
C_{rm} [-]	0.06	0%	0.12	5.1%
Steam [MJ]	4.5	40%	7.7	61%
Natural gas [MJ]	1.34	35%	2.0	36.9%
Electricity [MJ]	0.81	47.5%	1.07	54.5%
Cooling water [m^3]	0.104	30%	0.145	41.2%
Process water [m^3]	0.00065	35%	0.00159	42.5%
Direct emissions [$kg_{CO2}eq$]	0.43	72.5%	0.41	67.8%
Utilities_sum [$kg_{CO2}eq$]	0.89	45%	1.14	50.9%

The performance on the test data is slightly better than the results of the leave-one-out cross validation (Table S2) which strongly indicates that no overfitting on the LOO-CV data occurred. Thus, it is not expected that the final model performance will be overestimated by LOO-CV.

We use the tree depth and features as defined for the reduced training set and train the final trees on all 409 processes. The performance of these final trees is checked based on the leave-one-out validation approach. For this purpose, we trained 409 decision trees on 408 processes to predict each missing process. Following this strategy, we combine the benefits of having a set of independent test data and full utilization of all available data for the final model.

Feature reduction

We identified unnecessary features by calculating their importance for each of the trees. The relevance of a feature depended heavily on the output parameter estimated with the decision tree. Thus, features were only excluded if they were unnecessary for all trees (e.g., the amount of sulfur atoms in the stoichiometric equation). We further reduced the features by deleting groups of features systematically and checking whether the overall performance decreased significantly.

The performance is quantified by calculating the total decrease in impurity I_f for each feature f.[5, 6] The total decrease in impurity is calculated by summing up all weighted decreases in impurity i_{node} for nodes that use feature F as the decision criterion. The weighted decrease in impurity i_{node} is calculated for each node by subtracting the weighted mean absolute errors of each child node ($MAE_{node,l}$ and $MAE_{node,r}$) from the mean absolute error of the parent node (MAE_{node}) (Eq. S1).

$$i_{node} = \frac{N_t}{N} * \left(MAE_{node} - \frac{N_{t,l}}{N_t} * MAE_{node,l} - \frac{N_{t,r}}{N_t} * MAE_{node,r}\right) \qquad \text{Eq. S1}$$

N is the total number of samples. N_t is the number of samples at the current node, $N_{t,l}$ is the number of samples in the left child, and $N_{t,r}$ is the number of samples in the right child. See Figure S2 for the total decrease of impurity of the final decision trees.

This way, the reaction enthalpy, entropy and free enthalpy, and the melting points were excluded from the final set of features. The overall decrease of the performance after excluding these features was less than 3% for each of the trees. We believe this minor loss of performance is acceptable considering that the model could be simplified, which reduces the efforts for users and further limits overfitting. We further checked the performance when adding information about the temperature and pressure of the reaction. While that information is often unavailable at an early stage of research, the performance remained constant.

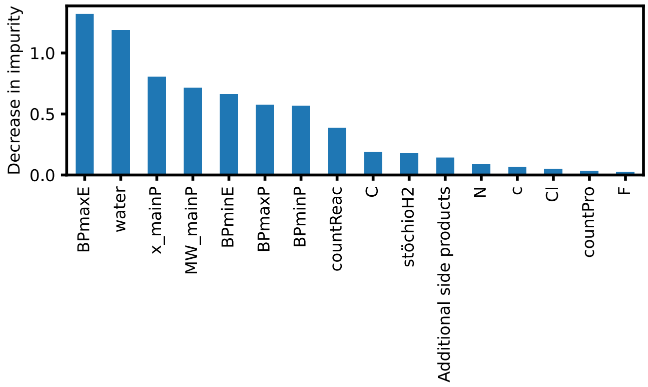

Figure S2 Cumulated feature importance (decrease in impurity I_f) for all decision trees used in the final model.

Even small decreases in impurity can be relevant for single trees and thus have not been excluded from the model. A substantial decrease in impurity suggests a high importance for the decision tree. The most important features are expected to strongly correlate with purification steps (e.g., boiling points, the amount of water formed during the reaction step, and the molar fraction of the main product). The maximum boiling point of reactants and the amount of water formed are central for the purification and reaction step, and consequently, they result in the highest decrease in impurity.

Additional side-products

The feature 'Additional side products' reflects whether side products are separated in the training data. In that case, side reactions lead to marketable side products being separated during the downstream process. The additional downstream efforts are thus included in the overall process energy demands. This information may not be available at an early stage of research. However, the decision trees can be used to estimate whether separating a side product might be preferable. For this purpose, both results (with and without additional side-products) can be calculated by altering the input. Generally, we recommend setting this value to 0 (no side-product), as the separation of side-products cannot be taken for granted.

This feature is used as a decision criterion in the decision trees for cooling water, electricity, C_{rm}, and steam (in descending order of feature importance). The estimates for natural gas, direct emissions, and process water are independent of the information about side products. When using the short decision trees shown below, the feature is only used for estimating the cooling water demand.

Performance of the short decision trees

Table S3 lists the performance criteria for estimating the overall GWI, the raw materials coefficient C_{rm}, direct emissions, and the sum of all utilities. The performance of the decision trees is compared to the best available proxies, and the relative improvements are provided. While we focus on the summed GWI of all utilities here, the performances for each separate utility are listed in Table S4.

Table S3: Performance measures for the mean absolute error (MAE) and the percentage of processes outside the AACE target range (-50% to + 100% of the actual GWI).

	Best available proxies		Decision trees			
Measure	MAE	Out of target range	MAE	Out of target range	Improvent for MAE	Improvement for target range
Overall $[\frac{kg_{CO2eq}}{kg_{main\,product}}]$	1.85	19.1%	1.46	11.0%	21.2%	42.4%
Raw materials coefficient C_{rm}	0.139	5.1%	0.105	3.7%	24.4%	28.1%
Direct emissions $[\frac{kg_{CO2eq}}{kg_{main\,product}}]$	0.501	87.3%	0.401	59.9%	20.0%	31.4%
$Utilities_{sum}$ $[\frac{kg_{CO2eq}}{kg_{main\,product}}]$	1.21	56.0%	1.03	45.5%	14.7%	18.8%

Table S4: Comparison of the final decision trees (all 409 processes used for training) with the previous best available proxy values [3] based on leave-one-out validation. The full trees have a depth of 7. The short trees have a depth of 4. The energy demands of the previous method are based on the averaged values reported by Kim & Overcash.[7] Their method does not account for natural gas, cooling water, and process water. Thus, no comparison was possible. In those three target categories, the short trees show a slightly better performance than the long trees due to the high number of processes without a demand for natural gas, cooling water, or process water. However, these demands are of minor importance to the overall results. MAE = mean absolute error, 'Out of AACE'= relative amount of processes that are not estimated within the range of -50% to +100% of the actual global warming impact as implied by the Cost estimate Guide.[1, 2]

	Best available proxies		Decision trees				Decision trees (short)			
Measure	MAE	Out of AACE	MAE	Out of AACE	Improvement		MAE	Out of AACE	Improvement	
					MAE	AACE			MAE	AACE
Total	1.9	19%	1.5	11%	21%	42%	1.6	15%	14%	19%
C_{rm}	0.14	5%	0.11	4%	24%	28%	0.12	4%	17%	28%
Steam [MJ]	9.4	64%	7.5	53%	20%	17%	8.3	62%	12%	3%
Natural gas [MJ]	NaN	NaN	2.0	41%	NaN	NaN	1.8	39%	NaN	NaN
Electricity	1.15	67%	1.06	54%	8%	19%	1.10	56%	5%	15%
Cooling water	NaN	NaN	0.14	39%	NaN	NaN	0.14	37%	NaN	NaN
Process water	NaN	NaN	1.5E-3	35%	NaN	NaN	1.4E-3	36%	NaN	NaN
Direct emissions	0.50	87%	0.40	60%	20%	31%	0.40	62%	21%	29%
$Utilities_{sum}$	1.2	56%	1.0	45%	15%	19%	1.1	54%	7%	3%

Table S4 compares the full decision trees (depth =7) and the short version (depth = 4) to the best available proxies identified in our previous study. The short decision trees already provide more accurate results compared to proxy values. Figures S3 and S4 visualize the performance of the short decision trees. A direct comparison between Figures S3 and S4 and Figures 2 and 3 indicates that the first four decisions are sufficient to capture most of the processes, while the following three layers further reduce the number of outliers and thus sharpen the distribution.

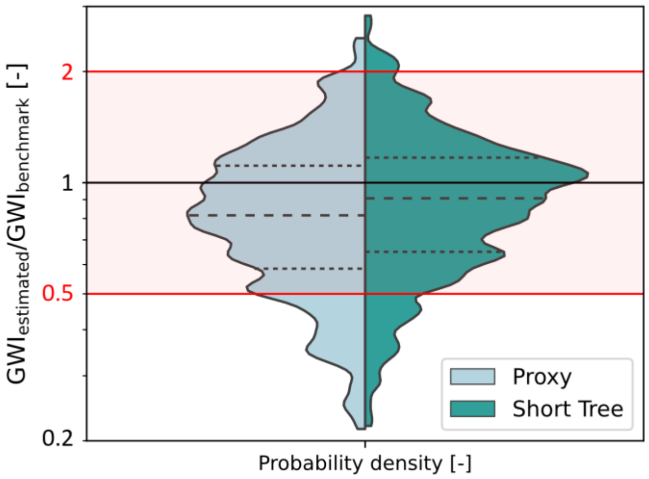

Figure S3: Violin plot comparing the overall GWI estimation performance when using the best available proxy values [3] for each technical flow (left) with the short decision trees (right). The short decision trees have a depth of 4 and are presented in the following. The performance is measured using a leave-one-out cross-validation. The sample lies on the black horizontal line if the estimated GWI is equal to the real GWI. Below this line, the GWI is underestimated; above, it is overestimated. The logarithmic scale shows the equal relevance of under- and overestimating by a factor of 2 (red lines). The red area between those red lines marks the range expected for cost estimates. The dashed lines in the violin plots mark the quartile ranges and the median of the data sets.

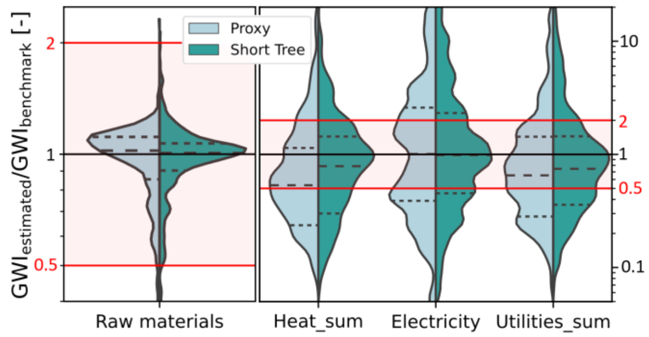

Figure S4 Violin plots comparing the estimation performance of the best available proxies (left) with the short decision trees (right) for estimating the aggregate GWI of the raw materials, the total heating demands in MJ, electricity demand in MJ and the aggregate GWI of all utilities. The short decision trees have a depth of 4 and are presented in the following. Please note the different ranges on the y-axis. The performance is measured using a leave-one-out cross-validation. The sample lies on the black horizontal line if the estimated value equals the process data. Below this line, the technical flow is underestimated; above, it is overestimated. The logarithmic scale shows the equal relevance of under- and overestimating by a factor of 2 (red lines). The red area between those red lines marks the range expected for cost estimates. The dashed lines in the violin plots mark the quartile ranges and the median of the data sets.

Short trees

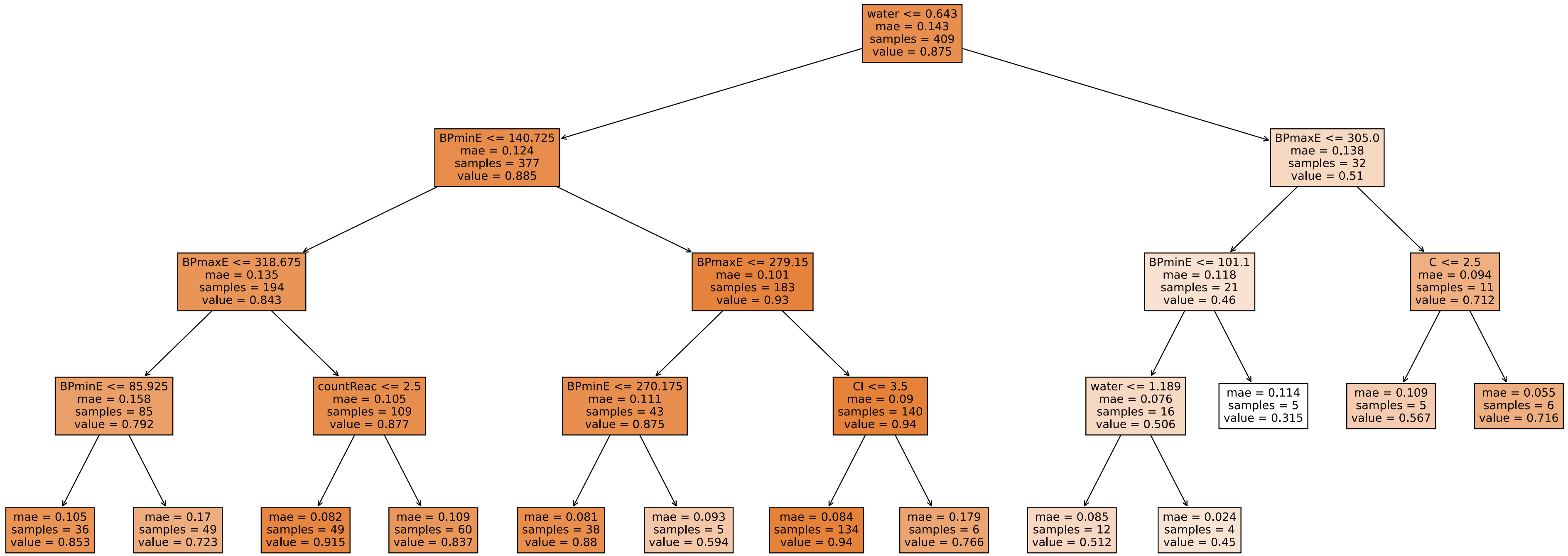

Figure S5 Short decision tree (first four decisions) for C_{rm}. Arrows to the left are for "true", and if the decision criterium is not met ("false"), the arrow goes to the right side. Abbreviations used: BP=boiling point, E=reactants, P=products, water=mass of water formed during the reaction per mass of main product, countReac=number of reactants, countPro=number of products, MW_mainP=molecular weight of the main product, x_mainP=molar fraction of main product to all products, stoichH2=mol of H2 required according to reaction equation, mae=mean absolute error.

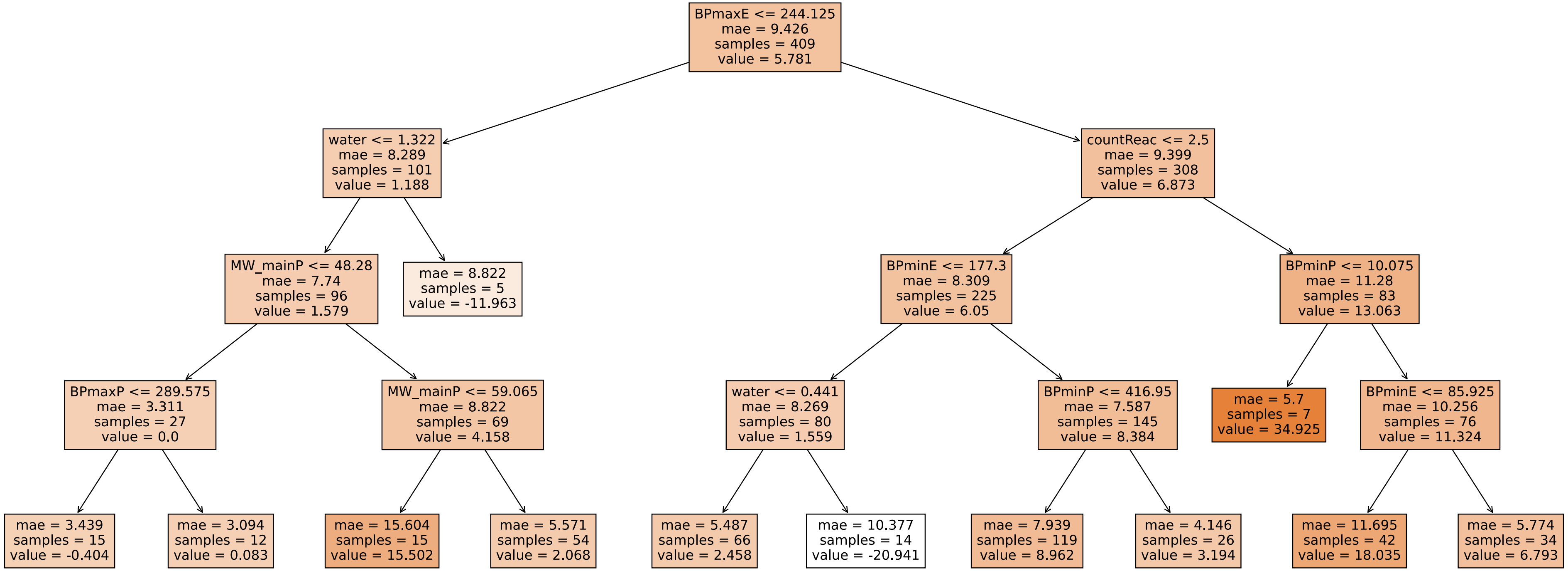

Figure S6 Short decision tree (first four decisions) for the **steam** demand in MJ. Arrows to the left are for "true", and if the decision criterium is not met ("false"), the arrow goes to the right side. Abbreviations used: BP=boiling point, E=reactants, P=products, water=mass of water formed during the reaction per mass of main product, countReac=number of reactants, MW_mainP=molecular weight of the main product, mae=mean absolute error.

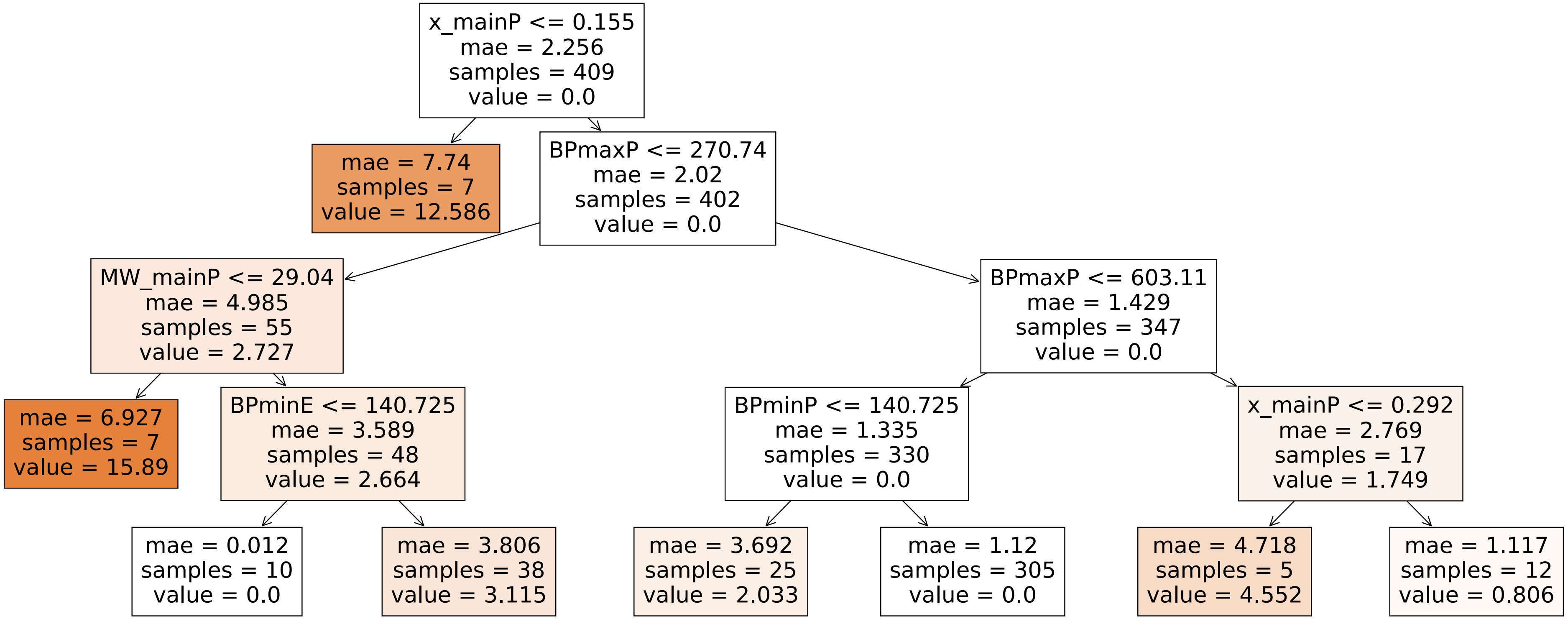

Figure S7 Short decision tree (first four decisions) for the **natural gas** demand in MJ. Arrows to the left are for "true", and if the decision criterium is not met ("false"), the arrow goes to the right side. Abbreviations used: BP=boiling point, E=reactants, P=products, MW_mainP=molecular weight of the main product, x_mainP=molar fraction of main product to all products, mae=mean absolute error.

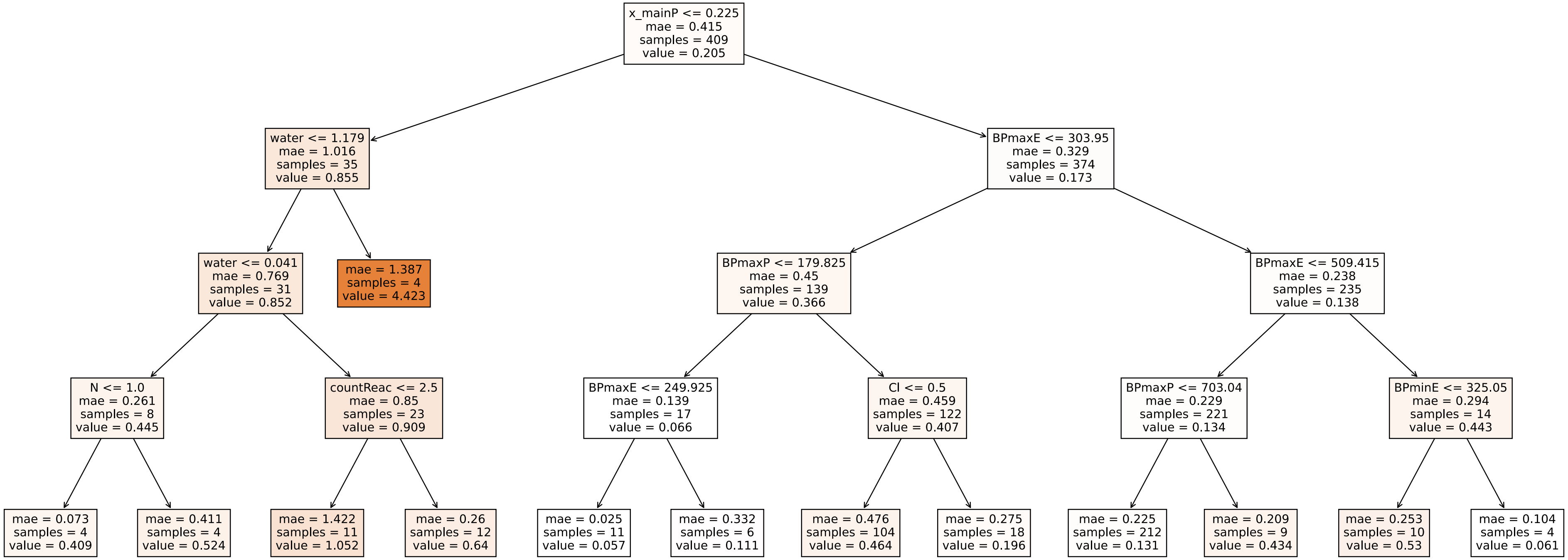

Figure S8 Short decision tree (first four decisions) for the **electricity** demand in MJ. Arrows to the left are for "true", and if the decision criterium is not met ("false"), the arrow goes to the right side. Abbreviations used: BP=boiling point, E=reactants, P=products, water=mass of water formed during the reaction per mass of main product, countReac=number of reactants, x_mainP=molar fraction of main product to all products, N/Cl= number of nitrogen/chlorine atoms in the reactants per mole of main product, mae=mean absolute error.

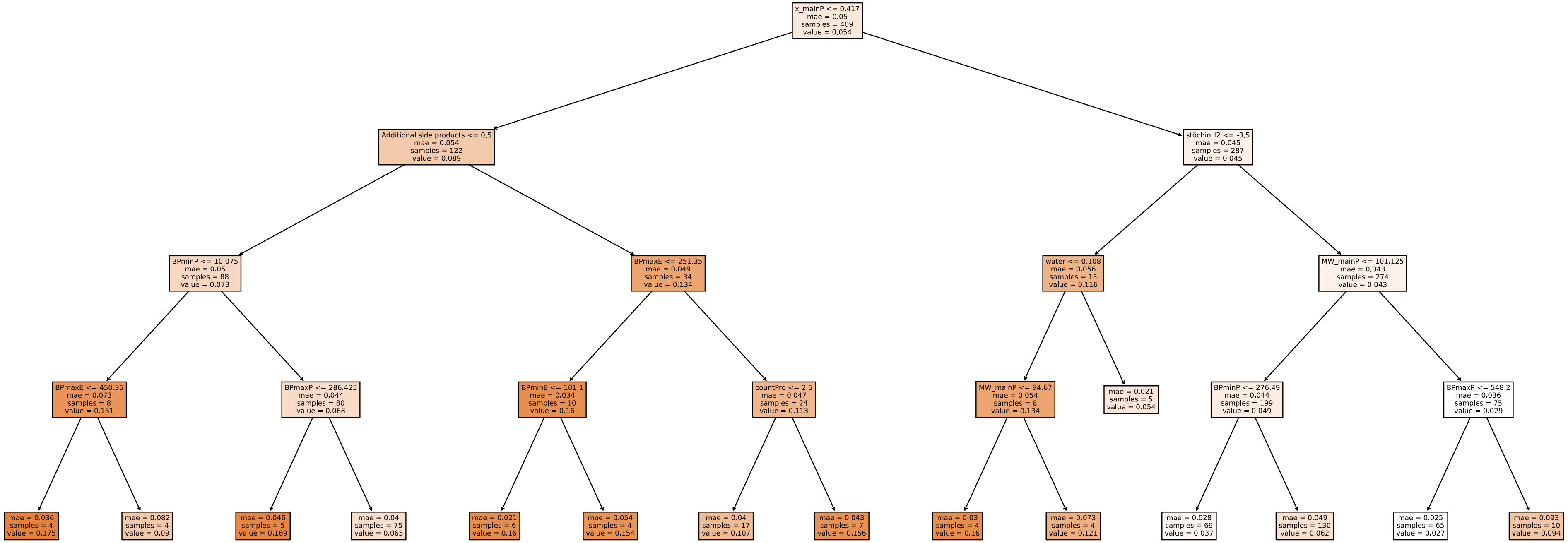

Figure S9 Short decision tree (first four decisions) for the **cooling water** demand in m^3. Arrows to the left are for "true", and if the decision criterium is not met ("false"), the arrow goes to the right side. Abbreviations used: BP=boiling point, E=reactants, P=products, water=mass of water formed during the reaction per mass of main product, countPro=number of products, MW_mainP=molecular weight of the main product, x_mainP=molar fraction of main product to all products, stoichH2=mol of H2 required according to reaction equation, mae=mean absolute error.

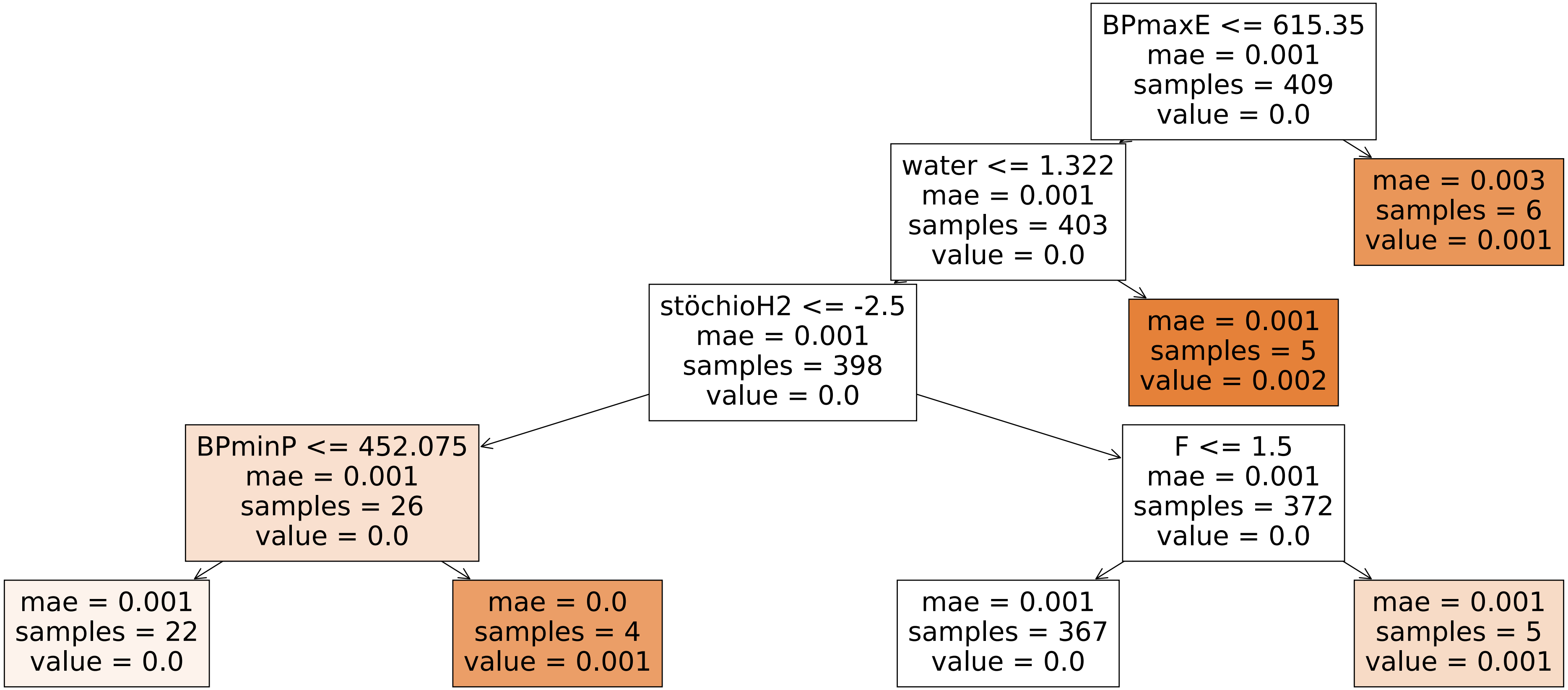

Figure S10 Short decision tree (first four decisions) for the **process water** demand in m^3. Arrows to the left are for "true", and if the decision criterium is not met ("false"), the arrow goes to the right side. Abbreviations used: BP=boiling point, E=reactants, P=products, water=mass of water formed during the reaction per mass of main product, stoichH2=mol of H2 required according to reaction equation, F=number of fluorine atoms in the reactants per mole of main product, mae=mean absolute error.

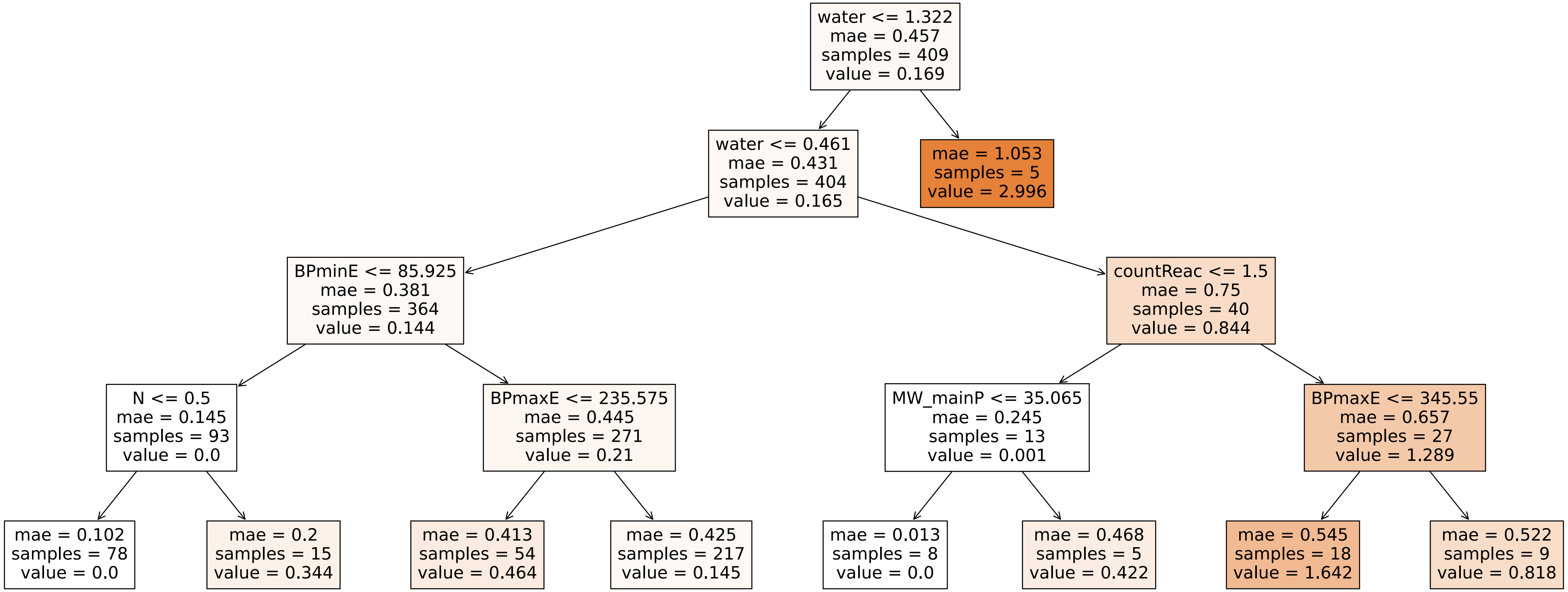

Figure S11 Short decision tree (first four decisions) for the **direct process emissions** in $kg_{CO2}eq$. Arrows to the left are for "true", and if the decision criterium is not met ("false"), the arrow goes to the right side. Abbreviations used: BP=boiling point, E=reactants, P=products, water=mass of water formed during the reaction per mass of main product, countReac=number of reactants, MW_mainP=molecular weight of the main product, N= number of nitrogen atoms in the reactants per mole of main product, mae=mean absolute error.

References

[1] L. R. Dysert and P. Christesen, "AACE International Recommended Practice No. 18R-97, Cost Estimate Classification System—As Applied in Engineering, Procurement, and Construction for the Process Industries," *Inc.: New York, NY, USA*, 2016.

[2] P. Christensen, L. R. Dysert, J. Bates, D. Burton, R. C. Creese, and J. Hollmann, "Cost Estimate Classification system-as applied in engineering, procurement, and construction for the process industries," *AACE, Inc*, vol. 2011, 2005.

[3] T. Langhorst, B. Winter, D. Roskosch, and A. Bardow, "Stoichiometry-Based Estimation of Climate Impacts of Emerging Chemical Processes: Method Benchmarking and Recommendations," *ACS Sustainable Chem. Eng.*, vol. 11, no. 17, pp. 6600–6609, 2023, doi: 10.1021/acssuschemeng.2c07624.

[4] IHS Markit, *Process Economics Program (PEP) Yearbook*: IHS Markit, London.

[5] F. Pedregosa *et al.,* "Scikit-learn: Machine learning in Python," *the Journal of machine Learning research*, vol. 12, pp. 2825–2830, 2011.

[6] L. Rokach and O. Maimon, "Decision trees," *Data mining and knowledge discovery handbook*, pp. 165–192, 2005.

[7] S. Kim and M. Overcash, "Energy in chemical manufacturing processes: gate-to-gate information for life cycle assessment," *J. Chem. Technol. Biotechnol.*, vol. 78, no. 9, pp. 995–1005, 2003, doi: 10.1002/jctb.821.
